# The spectrum of skin‐related conditions in primary care during 2015–2019–A Finnish nationwide database study

**DOI:** 10.1002/ski2.53

**Published:** 2021-06-05

**Authors:** A. Salava, A. Oker‐Blom, A. Remitz

**Affiliations:** ^1^ Department of Dermatology and Allergology Helsinki University Hospital Helsinki Finland

## Abstract

**Background:**

Skin‐related conditions are the frequent cause of doctors’ consultations in primary care.

**Methods:**

Based on nationwide data bank information of the Finnish Institute for Health and Welfare, we analysed the 20 most frequent main diagnoses for each ICD‐10 category of all general practitioners’ visits in the public health care in Finland over the years 2015–2019.

**Results:**

The total amount of doctor’s visits was 19 204 613 of which 1 489 228 consultations (7.80%) had a skin‐related condition as the main diagnosis. The most frequent skin‐related conditions were eczematous eruptions, bacterial skin infections and benign skin neoplasms accounting for 749 351 consultations (50.32%). The spectrum of skin‐related conditions was diverse, with a large quantity of rarer diagnoses. Some diagnoses showed significant proportional changes.

**Conclusions:**

The results demonstrate that a limited amount of conditions comprises most of the skin‐related consultations in primary care in Finland. Undergraduate education in dermatology should concentrate on the most frequent conditions seen by general practitioners, but also address the wide range of skin problems.

## Graphical abstract

The study provides a comprehensive picture about the spectrum of skin‐related conditions in primary care in Finland. Skin infections, eczematous eruptions and benign skin neoplasms are most frequently seen by general practitioners, but the range of skin problems is wide. Dermatology training should both concentrate on frequent conditions but also address the great variety of skin‐related conditions.

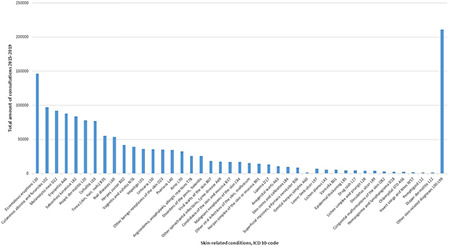

1

**What is already known about this topic?**

- Skin‐related conditions are frequent in primary care. Skin infections, eczematous eruptions and benign skin neoplasms seem to dominate, but there is a limited amount of prevalence data available.

**What does this study add?**

- The study provides a comprehensive picture about the spectrum of skin‐related conditions in primary care in Finland.
- Undergraduate dermatology training should both concentrate on frequent conditions but also address the great variety of skin‐related conditions.

## 2 INTRODUCTION

Skin‐related conditions are frequent consultation causes in primary care.^1^ ^,^ ^2^ Regardless of the geographical location or structure of the health care system, skin‐related conditions are among the most frequent diagnoses of general practitioners’ visits.^3^ ^,^ ^4^ A limited amount of diagnoses seems to dominate, but the spectrum of skin‐related conditions is wide.^5^ Based on nationwide database information, we aimed to investigate the most frequent skin‐related conditions seen by general practitioners in Finland and analyse their proportional changes over the years 2015–2019. In addition, we wanted to evaluate what proportion skin‐related conditions account for in primary care. In light of these results, we wanted to discuss if undergraduate training in dermatology concentrates on the correct conditions and if resources are directed adequately to dermatology compared with other medical specialities.

## 3 MATERIALS AND METHODS

We analysed the data bank of the Finnish Institute for Health and Welfare regarding outpatient doctors’ visits of the years 2015–2019 comprising most of the Finnish public primary health care sector.^6^ The year 2020 was not included because of possible bias based on the ongoing COVID‐19 pandemic and its effects on primary care. The data bank includes a main diagnosis for every doctor’s visit and has been digitally collected since 2015. The health care of Finland consists of a decentralised three‐level state‐funded health care system and a markedly smaller private sector. Its basic components are a large public primary care and the secondary and tertiary specialist health care (central and university hospitals). Doctor’s consultations in primary care are open to all citizens and free of charge. Primary care doctors are able to consulate the state organised specialist care or send the patient for specialist referral.

Search criteria for the analysis were doctor’s visit (main cause or diagnosis of actual consultation) and outpatient visit in primary care (mostly health centre). All age groups (0–99 years) and both sexes were included in the database search. Results for all Finnish municipalities were included. We searched for skin‐related conditions based on ICD‐10 classification codes: Diseases of the skin and subcutaneous tissue (L00‐L99), skin related infections (A00.0‐B99.9), skin neoplasms (D22, D23, D17, D18, C43‐C44) and other skin‐related diagnoses (e.g. congenital malformations Q82). The investigated database includes the 20 most frequent diagnoses for each ICD‐10 category (e.g., category L00‐L99). The remaining more infrequent diagnoses are grouped under ‘other diagnoses’. Subgroup diagnoses (i.e., L20.0) are not distinguished. Presented numbers signify absolute amounts of consultations in which a given diagnosis was the main cause of the general practitioner’s visit.

The proportional changes of the skin‐related conditions were analysed with the IBM SPSS Statistics 25.0 program. Differences in proportions were compared with the z‐test and p values < 0.05 were considered statistically significant.

## 4 RESULTS

### 4.1 Characteristics of the database search

During 2015–2019, the total amount of doctor’s visits with documented diagnoses was 19 204 613. There was a larger female gender prevalence: 11 137 588 (57.99%) female consultations and 8 067 025 (42.00%) male consultations.^7^ The amount of consultations was very similar throughout the investigated period with no substantial variation: 3 842 094 consultations in 2015, 3 855 140 consultations in 2016, 3 901 747 consultations in 2017, 3 911 512 consultations in 2018 and 3 694 120 in 2019 (Table 1).

**TABLE 1:** Amount of doctors’ visits in the public primary health care sector in Finland

Year	2015	2016	2017	2018	2019	2015–2019 (Cumulative)
Total amount of doctor’s visits (consultations)	3 842 094	3 855 140	3 901 747	3 911 512	3 694 120	19 204 613
Females	2 227 577	2 235 788	2 265 699	2 266 466	2 142 058	9 209 588
Males	1 614 517	1 619 352	1 636 048	1 645 046	1 552 062	8 067 025
Skin‐related condition as the main diagnosis of the consultation (% of all consultations)	291 881 (7.60%)	297 058 (7.71%)	298 826 (7.66%)	308 374 (7.88%)	293 089 (8.05%)	1 489 228 (7.80%)

Of the total amount of doctors’ visits, there were 1 489 228 (7.80%) consultations with a skin‐related condition. The 20 most frequent skin‐related conditions were the main diagnosis in 1 113 896 consultations, comprising 74.50% of doctors’ visits with a skin‐related condition. There was a significant variation of diagnoses and the spectrum was diverse, with a large quantity of rarer diagnoses. The database included information of different ICD‐10 categories and in total, there were 42 diagnoses recognised as skin‐related conditions. These accounted for 1 278 113 consultations (85.82%). In addition, there were 211 115 consultations (14.18%) with a diagnosis classified as ‘other skin‐related conditions’. This group was not differentiated more in the databank, but is likely to include important dermatological problems such as contact dermatitis or rosacea.

### 4.2 Amount and spectrum of skin‐related conditions

**FIGURE 1:**
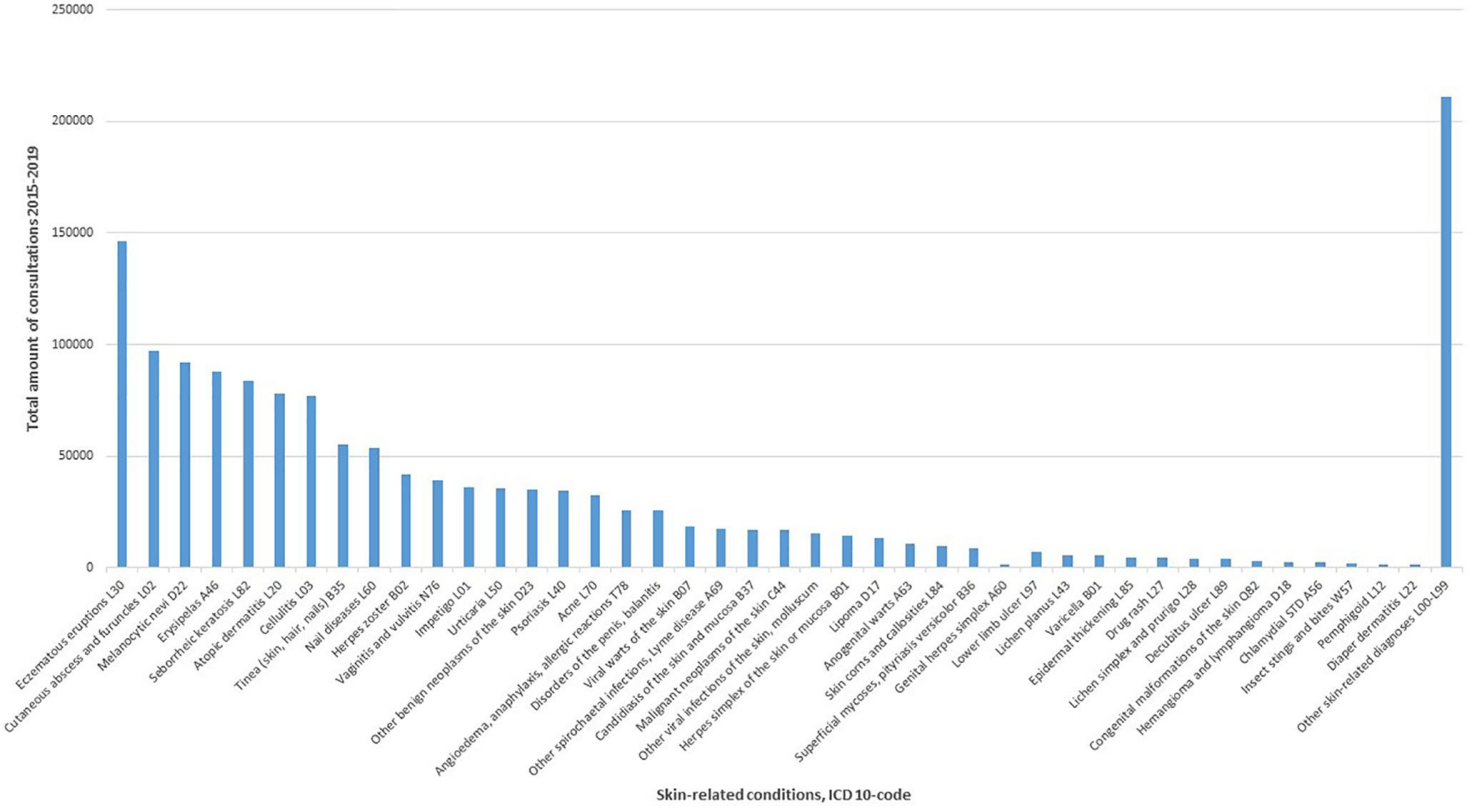
Amount of skin‐related conditions as main diagnosis of doctors’ visits in primary care in Finland during 2015–2019 (documented main diagnosis of single consultations)

The most frequent skin‐related conditions were eczematous eruptions (excluding atopic and contact dermatitis), cutaneous abscess, furuncle and carbuncle (L02), melanocytic nevi (D22) and erysipelas (A46) (Table 2, Figure 1).

**TABLE 2:** Doctors’ visits with skin‐related conditions (main reason of consultation)

Skin‐related diagnosis and ICD‐10 code	2015	2016	2017	2018	2019	2015–2019 (Cumulative)
Eczematous eruptions, dermatitis L30	28 877	28 405	29 273	30 466	29 313	146 334
Cutaneous abscess, furuncle and carbuncle L02	18 288	19 314	19 655	20 276	19 516	97 049
Melanocytic nevi D22	17 404	19 391	19 855	17 643	17 720	92 013
Erysipelas A46	16 549	17 483	17 234	19 276	17 315	87 857
Seborrhoeic keratosis L82	14 656	16 765	16 952	17 536	17 834	83 743
Atopic dermatitis L20	15 933	15 694	16 014	15 958	14 746	78 345
Cellulitis L30	16 433	14 927	14 587	15 316	15 547	76 810
Tinea (skin, hair and nails) B35	11 124	10 891	10 776	11 932	10 635	55 358
Nail diseases L60	9335	10 019	10 656	12 245	11 362	53 617
Herpes zoster B02	8210	8068	8339	8603	8685	41 905
Vaginitis and vulvitis N76	8028	8232	7805	7649	7687	39 401
Impetigo L01	7190	7209	6918	7976	7068	36 361
Urticaria L50	7176	6924	7486	7504	6580	35 670
Benign neoplasms of the skin D23	6548	6898	7545	7044	6876	34 911
Psoriasis L40	6777	6834	7206	6984	6697	34 498
Acne L70	6297	6379	6636	6886	6361	32 559
Angioedema and allergic reactions T78	6456	5914	4880	4828	3614	25 692
Disorders of the penis N48	5033	4919	5123	5359	5213	25 647
Viral warts B07	3853	3634	3725	3711	3560	18 483
Lyme disease A69	3657	3033	3367	3789	3797	17 643
Candidiasis B37	3522	3627	3363	3466	3125	17 103
Malignant neoplasms of the skin C44	3313	3550	3395	3329	3236	16 823
Viral infections of the skin B08	2997	3676	3034	3688	2347	15 742
Herpes simplex (skin and mucosa) B00	2733	2783	2970	3015	2841	14 342
Lipoma D17	2734	2785	2643	2632	2517	13 311
Anogenital warts A63	2176	2078	2184	2459	2198	11 095
Corns and callosities L84	2123	1968	1960	1981	1794	9826
Pityriasis versicolour B36	1716	1792	1857	1774	1562	8701
Genital herpes A60	1529	1391	1526	1473	1550	7469
Lower limb ulcer (unclassified) L97	1291	1347	1520	1711	1600	7469
Lichen planus L43	1140	1159	1214	1193	1165	5871
Varicella B01	1758	1694	1301	602	281	5636
Epidermal thickening L85	963	911	939	895	929	4637
Drug eruptions L27	921	933	955	901	832	4542
Lichen simplex and prurigo L28	830	934	882	885	827	4358
Decubitus ulcer L89	807	821	842	839	767	4076
Congenital malformations of the skin Q82	796	583	641	611	672	3303
Haemangioma and lymphangioma D18	459	565	553	505	535	2617
Chlamydial STI A56	662	474	391	406	445	2378
Insect stings and bites W57	1259	694	108	121	53	2235
Pemphigoid L12	248	243	229	289	370	1379
Diaper dermatitis L22	274	291	260	263	216	1304
Other skin related diagnoses	39 806	41 826	42 027	44 355	43 101	211 115

The group of eczematous eruptions with 146 334 consultations (9.83%), did not include irritant (L24) or allergic (L23) contact dermatitis, which both were not among the 42 detected skin‐related conditions. The most frequent skin infections were cutaneous abscesses, furuncle and carbuncle (L02) with 97 049 consultations (6.52%), erysipelas (A46) with 87 857 consultations (5.90%) and cellulitis (L03) with 76 810 consultations (5.16%). In addition, tinea (55 358 consultations, 3.72%), zoster (41 905 consultations, 2.81%) and impetigo (36 361 consultations, 2.44%) were frequently documented. Other frequent skin‐related infections included viral warts (18 483, 1.24%), Lyme disease (17 643, 1.18%), candidiasis (17 103, 1.15%), other viral infections of the skin and mucous membranes, for example molluscum contagiosum (15 742, 1.06%) and herpes simplex (14 342, 0.92%).

Benign skin neoplasms were a frequent cause of doctors’ consultations: melanocytic nevi (92 013 consultations, 6.18%), seborrhoeic keratosis (83 743, 5.62%) and other benign skin neoplasms (34 911, 2.34%). Malignant neoplasms of the skin were the main diagnosis in 16 823 doctor’s visits (1.13%). This group did not include cutaneous melanoma (C43) which was not under the 20 most frequent diagnoses in the category C00‐D49. Nail diseases were among the most frequent consultation causes (53 617, 3.60%). The group includes ingrown toenail, nail changes and dystrophy, but not paronychia (L03.0) which is included in the cellulitis diagnosis code.

In addition, other frequent skin‐related conditions included genital problems vulvitis and vaginitis (39 401 consultations, 2.65%) and disorders of the penis, including balanitis (25 647 consultations, 1.72%), urticaria (35 670, 2.39%), psoriasis (34 498, 2.32%), acne (32 559, 2.19%) and angioedema, allergic reactions and anaphylaxis (25 692, 1.73%). The most frequent causes of chronic leg ulcer are grouped in different ICD‐10 codes and include atherosclerosis (I70), venous stasis (I83), diabetes (E10‐11) and unclassified leg ulcer (L97). The amount of consultations due to atherosclerosis, venous stasis and diabetes was high, but based on the database information, the proportions associated with skin related conditions (e.g., ulcers and other skin problems) could not be determined. Unclassified lower limb ulcer accounted for 7469 consultations (0.50%) and decubitus ulcer L89 for 4076 consultations (0.27%).

### 4.3 Diagnoses grouped together

**FIGURE 2:**
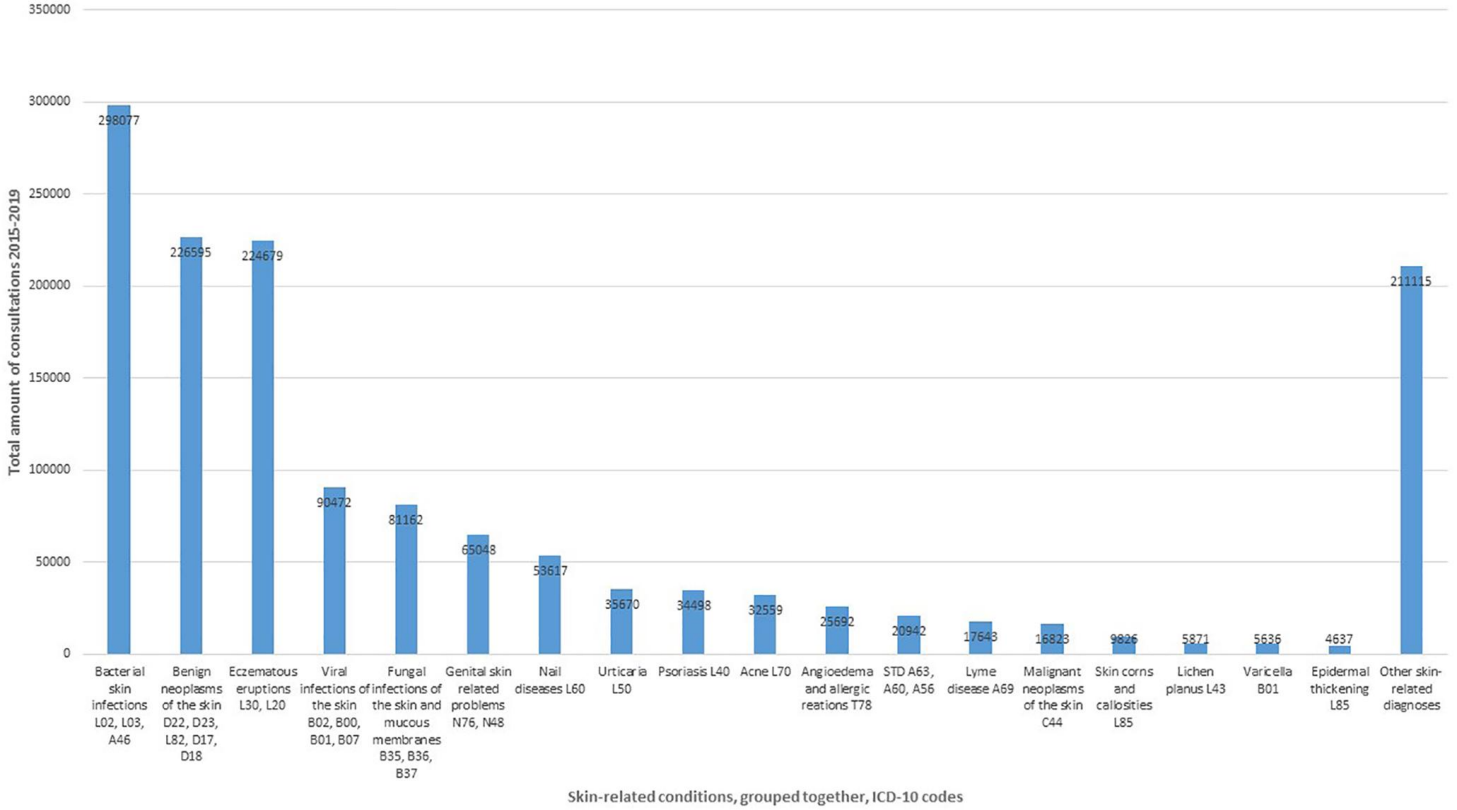
Diagnoses grouped together; amount of skin‐related conditions as main diagnosis of doctors’ visits in primary care in Finland during 2015–2019 (documented main diagnosis of single consultations)

Grouped together, the most frequent diagnosis groups where bacterial skin infections (298 077, 20.01%), eczematous eruptions including atopic dermatitis (224 679, 15.09%) and benign skin neoplasms (226 595 consultations, 15.22%) (Table 3, Figure 2). During 2015–2019, these three most frequent diagnosis groups accounted for 749 351 consultations, that is 50.32% of all doctors’ visits with skin‐related conditions. Grouped together, bacterial, viral and fungal skin infections were the most frequent group of diagnoses with 469 711 consultations (31.54%).

**TABLE 3:** Amount of doctors’ visits with the most frequent skin‐related conditions grouped together

Skin‐related diagnosis and ICD‐10 Code	2015–2019 (Cumulative)
Bacterial skin infections L01, L02, L03, A46	298 077
Eczematous eruptions L30, L20	224 679
Benign neoplasms of the skin D22, D23, L82, D17, D18	226 595
Viral infections of the skin B02, B00, B01, B07	90 472
Fungal infections of the skin and mucous membranes B35, B36, B37	81 162
Genital skin related‐conditions N76, N48	65 048
Nail diseases L60	53 617
Urticaria L50	35 670
Psoriasis L40	34 498
Acne L70	32 559
Angioedema and allergic reactions T78	25 692
Chlamydial sexually transmitted infections A63, A60, A56	20 942
Lyme disease A69	17 643
Malignant neoplasms of the skin C44	16 823
Skin corns and callosities L85	9826
Lower limb ulcer (unclassified) L97	7469
Lichen planus L43	5871
Varicella B01	5636
Epidermal thickening L85	4637
Drug eruptions	4542
Lichen simplex and prurigo	4358
Decubitus ulcer L89	4076
Congenital malformations of the skin	3303
Insect stings and bites	2235
Pemphigoid	1379
Diaper dermatitis	1304
Other skin‐related conditions	211 115

### 4.4 Proportional changes of specific skin‐related conditions during 2015–2019

**FIGURE 3:**
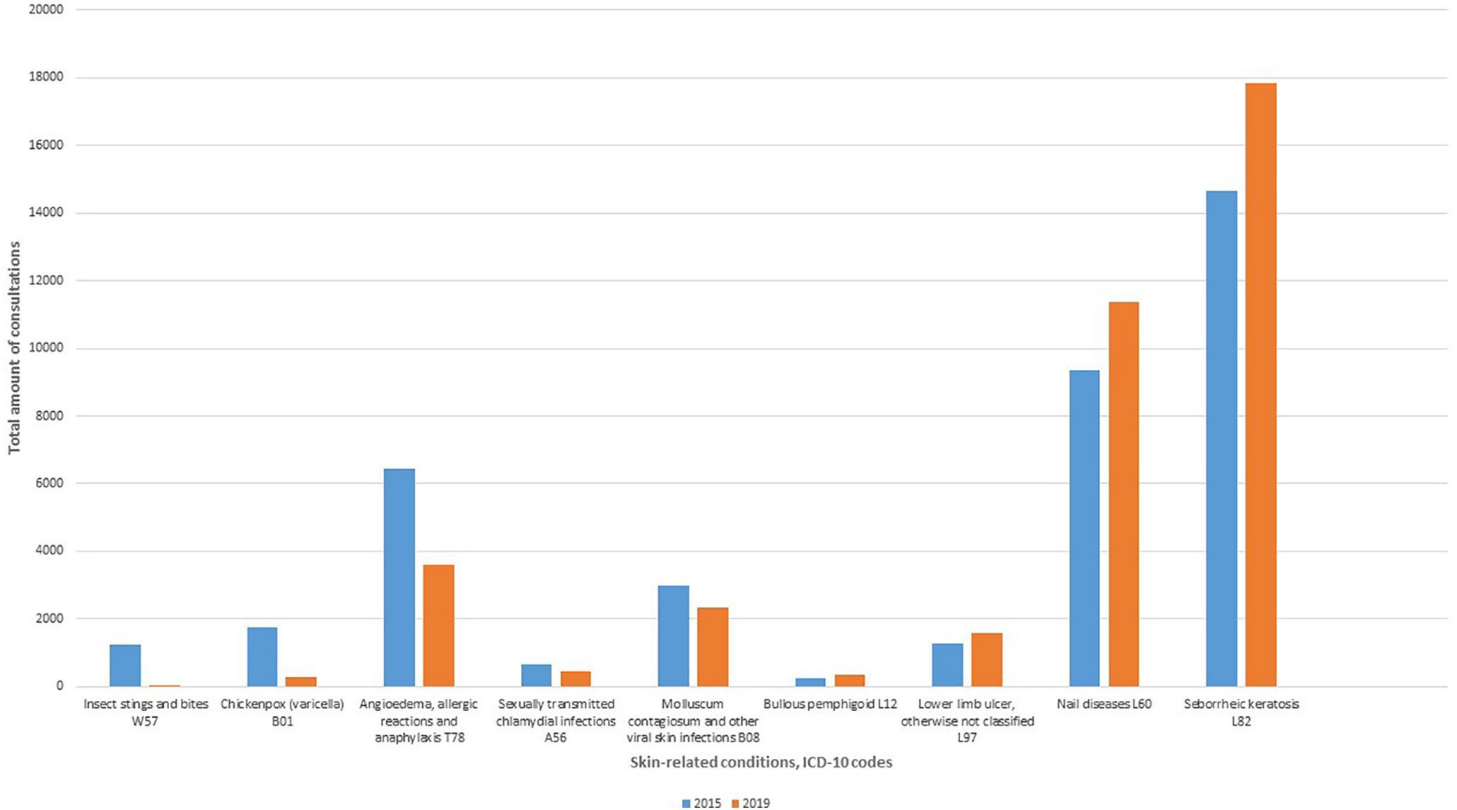
Skin‐related conditions with significant proportional changes during 2015–2019

Some diagnoses showed significant proportional changes. The proportions of the following skin‐related conditions decreased significantly during 2015–2019: Insect stings and bites (percentage change −95.8%, difference in proportion 0.00413, 95% CI 0.0039–0.0044, p < 0.001), varicella (percentage change −84.0%, difference in proportion 0.00506, 95% CI 0.0048–0,0054, p < 0.001), angioedema, allergic reactions and anaphylaxis (percentage change −44.0%, difference in proportion 0.00979, 95% CI 0.0091–0.0105, p < 0.001), sexually transmitted chlamydial infections (percentage change −32.8%, difference in proportion 0.00075, 95% CI 0.0005–0.001, p < 0.001), other viral infections of the skin, for example molluscum contagiosum (percentage change −21.7%, difference in proportion 0.00226, 95% CI 0.0018–0.0027, p < 0.001) (Figure 3).

On the contrary, proportions of the following skin‐related conditions increased significantly: pemphigoid (percentage change +49.2%, difference in proportion 0.00041, 95% CI 0.0002‐0.0006, p < 0.001), lower limb ulcer (percentage change +23.9%, difference in proportion 0.00103, 95% CI 0.0007–0.0014, p < 0.001), nail diseases (percentage change +21.7%, difference in proportion 0.00678, 95% CI 0.0058–0.0077, p < 0.001) seborrhoeic keratosis (percentage change +21.7%, difference in proportion 0.01064, 95% CI 0.0095–0.0118, p < 0.001). The most frequent skin‐related conditions and diagnoses grouped together (skin infections, eczematous eruptions and benign skin neoplasms) did not show significant proportional changes.

## 5 DISCUSSION

The results of this nationwide database study demonstrate that a limited amount of diagnoses comprises most of the skin‐related conditions in primary care in Finland. The most frequent being skin infections, eczematous eruptions and benign skin neoplasms.^8^ ^,^ ^9^ We think that undergraduate training in dermatology should concentrate on providing a strong basis of practical and theoretical knowledge of frequent skin‐related conditions.^10^ ^,^ ^11^ In addition, the database analysis shows the wide spectrum of skin‐related conditions, which should also be addressed in training of medical students.^12^ ^,^ ^13^

Schoefield et al. analysed surveillance data for 2006 and showed that skin conditions were the commonest new presenting reason to general practitioners in England and Wales.^1^ The study data revealed that skin‐related conditions accounted for 24% of all consultations. There was no evidence of increasing or decreasing trends during 2006. Kerr et al. investigated all skin‐related consultations during a 2‐week period in 13 selected general practices in Edinburgh and Lothian in Scotland.^5^ Similarly, skin‐related conditions were the most frequent cause of doctors’ visits, accounting for 18.8% of all consultations. In both studies, the most frequent were skin infections, eczematous eruptions and benign skin neoplasms, very similar to our study data. The relative amounts in our data were lower: skin‐related conditions accounted for 7.80% of doctors’ visits in primary care. The lower proportion compared to previous studies might be explained by the limited information of the investigated database in which only the main diagnosis was documented. The diagnosis of a patient´s major chronic disease, for example diabetes, is often documented as the reason of consultation in primary care and therefore the real amount of skin‐related conditions may be underestimated.^11^

Nevertheless, our understanding of the spectrum of skin‐related conditions in primary care remains uncomplete.^1^, ^3^, ^5^ It is likely to differ substantially from that of specialist care. Buendía‐Eisman et al. found that the most common diagnoses recorded by Spanish dermatologist in outpatient clinics were actinic keratosis, basal cell carcinoma and melanocytic nevus.^14^ González‐Cruz et al. investigated referrals from primary care to dermatologists and found that 31.6% were for cystic lesions or benign tumours and so potentially avoidable.^15^ Many patients (22%) could be discharged on the first visit; in these cases, the most frequent diagnoses were seborrhoeic keratosis (9.3%) and melanocytic nevus (8.6%). The results underline the importance of benign skin neoplasms in primary care, which was also observable in our data although we did not analyse referrals to specialist care.

Doctors’ visits in primary care usually include a wide range of problems and aim to acquire a holistic view of the patients’ health. Skin‐related conditions are often addressed alongside other complaints.^16^ Thus, they are more likely to be left undocumented as separate diagnoses. Salvi et al. found in a 1‐day point prevalence study in India, that skin‐related conditions were the third most frequent consultation cause in primary care (prevalence 9.0%) after gastrointestinal disorders (prevalence 25.0%) and cardio‐vascular disorders (prevalence 12.5%).^3^ Skin‐related conditions seem to be frequent in primary care independently of the geographical region or structure of the health care system.^17^

Our results show that the spectrum of skin‐related conditions in primary care in Finland is diverse. There were three most frequent diagnosis groups, a range of other frequent diagnoses, and in addition, a substantial amount of rarer, in the database not discriminated diagnoses. A total of 211 115 doctors’ visits had a rarer skin‐related condition, which accounted for 14.18% of all documented skin‐related consultations. Although this group could not be differentiated by the database information, it probably includes frequent dermatological problems of the general population, for example contact dermatitis (L23‐L25), hair diseases (L63‐L68) and rosacea (L71).

The proportions of the most frequent skin‐related conditions did not change significantly during 2015–2019. Significant changes seen in some ICD‐10 codes with relatively small total amounts might be based on normal fluctuation. The decrease of varicella is likely to be explained by the introduction of the varicella vaccination to the national vaccination program in autumn 2017.^18^ Decrease of the proportion of insect stings, angioedema and anaphylaxis, chlamydial STD and molluscum contagiosum may be caused by changes in documentation codes and habits. The increase of nail diseases and seborrhoeic keratosis might be linked to changes in demographic structure and ageing of the population. The prevalence of bullous pemphigoid^19^ and chronic lower limb ulcer^20^ seems to be increasing in developed countries, and our data shows this trend in the primary health care of Finland.

The main limitation of this study was classification bias. Diagnoses were not verified by dermatologists, and this could have led to classification bias and explain the lower proportions of relatively common diseases in dermatologic outpatient setting such as rosacea, contact dermatitis or hidradenitis suppurativa. In addition, the data includes only information about doctors’ visits, but not nurses’ or other health care specialists’ consultations, which can be extensive in primary care.^21^ Eczematous eruptions (L30) was the most frequent skin‐related diagnosis, but the amount of consultations might be overestimated, because often contact dermatitis is classified under eczematous eruptions (L30). In addition, the code L30.9 is frequently used to classify unknown or unspecific skin eruptions and not always consequently. Diagnosis codes for skin infections are frequently documented (bacterial, viral or fungal infections) without microbiological verification and other skin‐related conditions may have been classified as infections. We analysed data for the whole country and there might be differences in geographical distributions. A recognised limitation of the study was also, that skin‐related subgroup diagnoses (e.g., L30.0 for nummular dermatitis) could not be analysed.

The results of this nationwide database study show that three groups dominate the skin‐related problems seen by doctors’ in the Finnish primary health care. The study included a very large number of analysed doctors’ visits in the public primary health care sector and provides a comprehensive picture about the spectrum of skin‐related conditions that general practitioners encounter in Finland. The great variety of skin‐related conditions in primary care was also observable. The diversity of skin‐related conditions makes the diagnosis, differential diagnoses and management challenging, especially for doctors’ in training.^22^, ^23^, ^24^, ^25^ We therefore believe that it would be important in undergraduate teaching to both concentrate on frequent conditions but also address the wide range of skin‐related problems in primary care.^26^ ^,^ ^27^ Dermatology represents a small clinical speciality in medical school, and thus training of skin‐related conditions is often very limited.^28^ ^,^ ^29^ As a future perspective, we think that more studies are needed to address the question if resources of medical training are directed adequately to dermatology compared with other medical specialities.^30^ ^,^ ^31^

## CONFLICTS OF INTEREST

The authors declare that there is no conflict of interest.
